# Dramatic Increase in Oxidative Stress in Carbon-Irradiated Normal Human Skin Fibroblasts

**DOI:** 10.1371/journal.pone.0085158

**Published:** 2013-12-23

**Authors:** Carine Laurent, Alexandre Leduc, Ivannah Pottier, Virginie Prévost, François Sichel, Jean-Louis Lefaix

**Affiliations:** 1 SAPHYN (Santé Physique Nucléaire), ARCHADE (Advanced Resource Centre for Hadrontherapy in Europe) Program, Caen, France; 2 ABTE (Aliments, Bioprocédés, Toxicologie, Environnements), EA4651, Université de Caen-Basse Normandie, Caen, France; 3 CLCC (Centre de Lutte Contre le Cancer) François Baclesse, Caen, France; 4 INSERM (Institut National de la Santé et de la Recherche Médicale), U1086 Cancer et Préventions, Université de Caen-Basse Normandie, Caen, France; 5 LARIA (Laboratoire d’Accueil et de Recherche avec les Ions Accélérés), CEA (Commissariat à l’Energie Atomique) – DSV (Direction des Sciences du Vivant) – IRCM (Institut de Radiobiologie Cellulaire et Moléculaire), Caen, France; Kagoshima University Graduate School of Medical and Dental Sciences, Japan

## Abstract

Skin complications were recently reported after carbon-ion (C-ion) radiation therapy. Oxidative stress is considered an important pathway in the appearance of late skin reactions. We evaluated oxidative stress in normal human skin fibroblasts after carbon-ion *vs.* X-ray irradiation. Survival curves and radiobiological parameters were calculated. DNA damage was quantified, as were lipid peroxidation (LPO), protein carbonylation and antioxidant enzyme activities. Reduced and oxidized glutathione ratios (GSH/GSSG) were determined. Proinflammatory cytokine secretion in culture supernatants was evaluated. The relative biological effectiveness (RBE) of C-ions vs. X-rays was 4.8 at D_0_ (irradiation dose corresponding to a surviving fraction of 37%). Surviving fraction at 2 Gy (SF2) was 71.8% and 7.6% for X-rays and C-ions, respectively. Compared with X-rays, immediate DNA damage was increased less after C-ions, but a late increase was observed at D_10%_ (irradiation dose corresponding to a surviving fraction of 10%). LPO products and protein carbonyls were only increased 24 hours after C-ions. After X-rays, superoxide dismutase (SOD) activity was strongly increased immediately and on day 14 at D_0%_ (irradiation dose corresponding to a surviving fraction of around 0%), catalase activity was unchanged and glutathione peroxidase (GPx) activity was increased only on day 14. These activities were decreased after C-ions compared with X-rays. GSH/GSSG was unchanged after X-rays but was decreased immediately after C-ion irradiation before an increase from day 7. Secretion of IL-6 was increased at late times after X-ray irradiation. After C-ion irradiation, IL-6 concentration was increased on day 7 but was lower compared with X-rays at later times. C-ion effects on normal human skin fibroblasts seemed to be harmful in comparison with X-rays as they produce late DNA damage, LPO products and protein carbonyls, and as they decrease antioxidant defences. Mechanisms leading to this discrepancy between the two types of radiation should be investigated.

## Introduction

Effects of conventional radiation therapy (RT) using low-LET (linear energy transfer) X-rays on tumours and on normal tissues have been investigated for decades. Proton therapy, which is a more recent RT modality, has proven effective on tumours with a more precise dose delivery. C-ion therapy should have the same advantages with a higher RBE. Indeed, when proton RBE is considered as 1.15 compared with X-rays [1], the RBE of C-ions is estimated to 2 to 3 in tumours [2]. However, C-ion hadrontherapy is still underinvestigated, especially concerning normal tissues. Some *in vitro* studies showed no difference in RBE_10%_ of tumour cells *vs.* normal cells after C-ion irradiation [2]. Moreover, recent reports showed acute and late skin complications after C-ion RT [3]. Of 35 patients treated for unresectable bone and soft tissue sarcoma by a dose escalation protocol, 35 and 27 presented acute or late skin reactions, respectively, after exposure to doses ranging from 52.8 to 73.6 GyE in 16 fixed fractions. They were followed up from 29.5 to 71.7 months after C-ion RT. Late skin reactions reached grade IV (RTCOG/EORTC Scoring System). It was long considered that radiation-induced late cutaneous injury was only due to the delayed mitotic death of dermal parenchymal [4] or vascular cells, thus explaining that the lesions are progressive and inevitable. But *in vitro* studies have demonstrated an active role of dermal fibroblasts and endothelial cells in response to irradiation by the use of an anti-inflammatory and antioxidant treatments [5,6], which have proven very effective in patients presenting late skin complications [7]. 

Oxidative stress (OS) is an important pathway leading potentially to cell death after irradiation through oxidative damage to biological macromolecules when antioxidant defences are overwhelmed. While DNA is considered as the main target of radiation by direct or indirect effects, it is now thought that ROS (Reactive Oxygen Species) are greatly involved in cellular DNA and macromolecule damage as they are produced in early and late waves and are maintained over a long period of time after irradiation. Few studies have been performed on OS occurring after C-ion irradiation. Wan et al. [8] reported that peroxide production was similar in human epithelial cells after proton or X-ray irradiation, but was reduced after ^56^Fe ion irradiation. They observed that a selection of antioxidants delivered alone or in combination and administered either before or during irradiation protected MCF10 breast epithelial cells irradiated with X-rays, γ-rays, protons, or HZE (high Z and high energy) particles against OS [9]. *In vivo* studies were also performed with antioxidants given to rodents exposed to HZE particles vs. protons or γ-rays [10-13]. These compounds protected against OS as measured in plasma using the Total Antioxidant Status assay. Concerning C-ion irradiation especially, a study of gene regulation of the oxidative stress pathway *in vitro* showed an increase in heme oxygenase-1 and NAD(P)H dehydrogenase-quinone-1 after C-ion irradiation [14], but no X-ray irradiation was performed for comparative purposes. *In vivo*, gene regulation of mouse tumours transplanted in C3H/HeNrs mice was not modified after X-ray or C-ion exposure [15]. Taken together, these results tend to indicate that OS plays a major role after high-LET radiation.

In the present work, we were interested in human skin fibroblasts from young adult healthy individuals, as skin is the first organ exposed during RT. Cells were irradiated at confluence (G_0_) to mimic skin physiology, either with 5 MV X-rays or with 75 MeV/n C-ions corresponding to a real energy to cells of 72 MeV/n (LET = 33.6 keV/µm) [16]. This C-ion energy delivered at the GANIL facility (Caen, France) corresponds to the delivered dose in the plateau-phase before the Spread-Out Bragg Peak (SOBP). This was well adapted to our experiments as skin is exposed to this energy. OS parameters were measured until 21 days after X-ray or C-ion irradiation at D_10%_ and D_0%_ corresponding to ~10% and ~0% survival, respectively. Concerning C-ion irradiation, the doses corresponded to the range of 3.3 to 4.6 GyE/16 fractions delivered at NIRS (Chiba, Japan) when acute and late skin reactions were encountered [3]. 

## Materials and Methods

### Cell cultures

Primary cultures of normal human dermal fibroblasts (Lonza, Verviers, Belgium) were grown in DMEM supplemented with 20% FBS, 100 µg/mL L-glutamine, 10 mM HEPES and antibiotics (100 units/mL penicillin and 100 µg/mL streptomycin), at 37°C in a humidified atmosphere containing 5% CO_2_ and were used in passages 4-7. 

### X-ray and C-ion irradiation

Confluent cells were irradiated at room temperature either with X-rays using an Orion generator (CGR MEV, Riverside, CA, USA; 5 MV, 1.4 Gy/min) or with C-ions on the D1 line of the GANIL accelerator (Caen, France; 75 MeV/n, 1 Gy/min) [16]. C-ion irradiation was carried out in the LARIA facility during different runs between 2008 and 2011. Cells were kept until 21 days after irradiation and medium was replaced twice a week.

### Clonogenic survival

Eighteen hours after irradiation at confluence, cells were trypsinized. Clonogenic assessment was done according to the historical method described by Puck et al. [17]. Briefly, 1000 to 5000 cells were plated in 25 cm^2^ tissue culture flasks. Colonies ≥50 cells were scored 10-14 days after irradiation and the surviving fraction (SF) was calculated taking into account control cell plating efficiency. SF was fitted using the linear-quadratic model. For subsequent experiments, chosen irradiation doses were approximately D_10%_ and D_0%_ corresponding respectively to 2 and 6 Gy for C-ions and to 6 and 10 Gy for X-rays.

### Alkaline comet assay

The alkaline single-cell gel electrophoresis assay described by Laurent et al. [5] was used. The mean Olive Tail Moment (OTM) immediately, 1 hour or 3 hours after irradiation was calculated using the computer image analysis software Casp. 

### Malondialdehyde and 4-hydroxyalkenals in cell homogenates

Lipid peroxidation products were quantified using a Calbiochem assay kit. Cells were lysed by a thermal shock in [Tris/HCl 10 mM, Triton 0.1%, sucrose 200 mM] buffer. Malondialdehyde (MDA) and 4-hydroxyalkenals (HAEs) were measured by a colorimetric assay.

### Protein carbonyls in cell homogenates

 Protein carbonylation was measured using the Millipore OxyELISA^TM^ Oxidized Protein Quantitation kit. Cells were lysed by a thermal shock in [Tris/HCl 10 mM, Triton 0.1%, sucrose 200 mM] buffer. 

### Superoxide dismutase, catalase and glutathione peroxidase activities in cell homogenates

Total SOD, catalase and GPx activities were measured using Calbiochem assay kits as described by the manufacturer. Cells were lysed by a thermal shock in [Tris/HCl 10 mM, Triton 0.1%, sucrose 200 mM] buffer.

### Reduced and oxidized glutathione ratio in cell homogenates

 Reduced and oxidized glutathione ratios (GSH/GSSG) were measured using a Calbiochem assay kit as described by the manufacturer. Cells were lysed by a thermal shock in [Tris/HCl 10 mM, Triton 0.1%, sucrose 200 mM] buffer.

### Cytokine concentrations in supernatants

TNF-α, IL-6 and IL-1β in cell supernatants were quantified by means of a chemiluminescent enzyme immunometric assay employing an IMMULITE® 1000 automated analyser (Siemens Healthcare Diagnostics S.A.S). The sensitivity of the assay was 2, 4 and 5 pg/mL for IL-6, TNF-α and IL-1β, respectively. 

### Statistical analysis

Results were normalized to control values. Data are depicted as mean ± SEM. ** for *p*<0.001 or * for *p*<0.05 for X-irradiated cells compared with control cells and †† for *p*<0.001 or † for *p*<0.05 for C-ion compared with X-ray irradiated cells (one-way ANOVA with the Tukey test). Each experiment was done independently in triplicate.

## Results

### Survival

**Figure 1:**
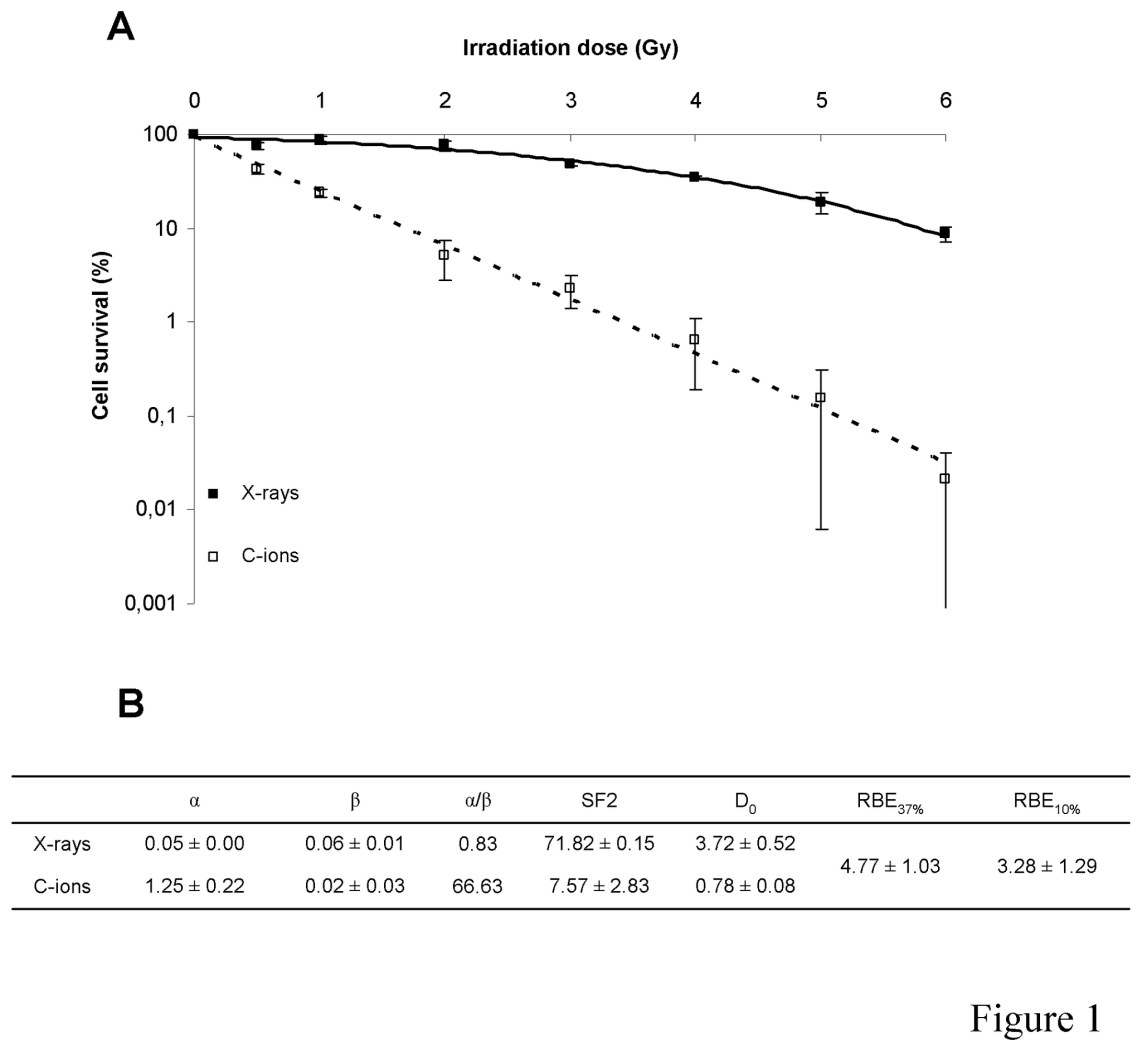
Fibroblast clonogenic survival after X-ray or C-ion irradiation. A: clonogenic survival curves; B: radiobiological parameters, α (Gy^-1^), β (Gy^-2^), SF2 (%), D_0_ (Gy).

Fibroblast survival fractions were greatly decreased after C-ion compared with X-ray irradiation (Figure 1A). SF2 and D_0_ values presented a 9.5-fold and a 4.6-fold decrease after C-ion vs. X-ray irradiation with 7.6% vs. 71.8% and 0.8 Gy vs. 3.7 Gy, respectively (Figure 1B). RBE values were 4.77 for 37% and 3.28 for 10% survival, respectively. The α value representing radiation sensitivity was 25-fold higher after C-ions compared with X-rays. In contrast, the β value was 3-fold lower after C-ions compared with X-rays, with a value of 0.02 explaining the almost linear shapes of the C-ion survival curve. The α/β ratio was increased 83-fold after C-ions compared with X-rays, with respective values of 66.6 and 0.8. D_10%_ and D_0%_ irradiation doses chosen for subsequent experiments corresponded to 6 Gy and 10 Gy for X-rays and 2 Gy and 6 Gy for C-ions, respectively.

### DNA damage

**Figure 2:**
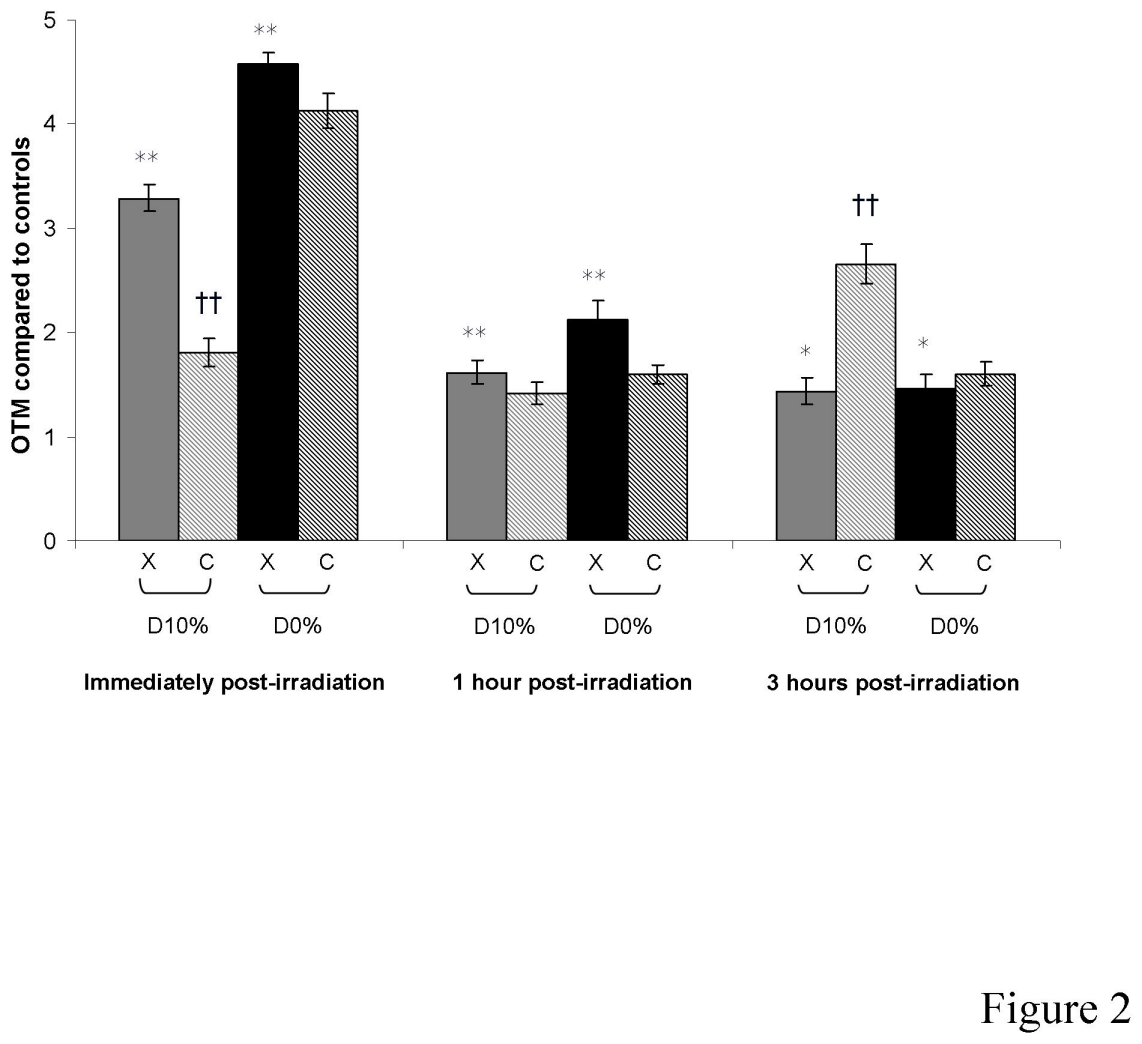
DNA damage measured by alkaline comet assay in fibroblasts irradiated by X-rays or C-ions. The mean olive tail moment of non-irradiated cells was 1.26 ± 0.17.

DNA single- and double-strand breaks as well as alkali-labile sites were quantified by means of the alkaline comet assay (Figure 2). OTMs were increased immediately after X-rays in a dose-dependent manner. Immediately after C-ion irradiation, OTMs were less increased than after X-rays, with an RBE_10%_ of DNA damage induction of 0.62. One hour after irradiation, OTMs returned to control levels for C-ion irradiated but not for X-irradiated fibroblasts. At 3 hours, a new increase in OTMs occurred only in C-ion irradiated fibroblasts, with an RBE_10%_ of 2.80.

### Lipid peroxidation and protein carbonylation products

**Figure 3:**
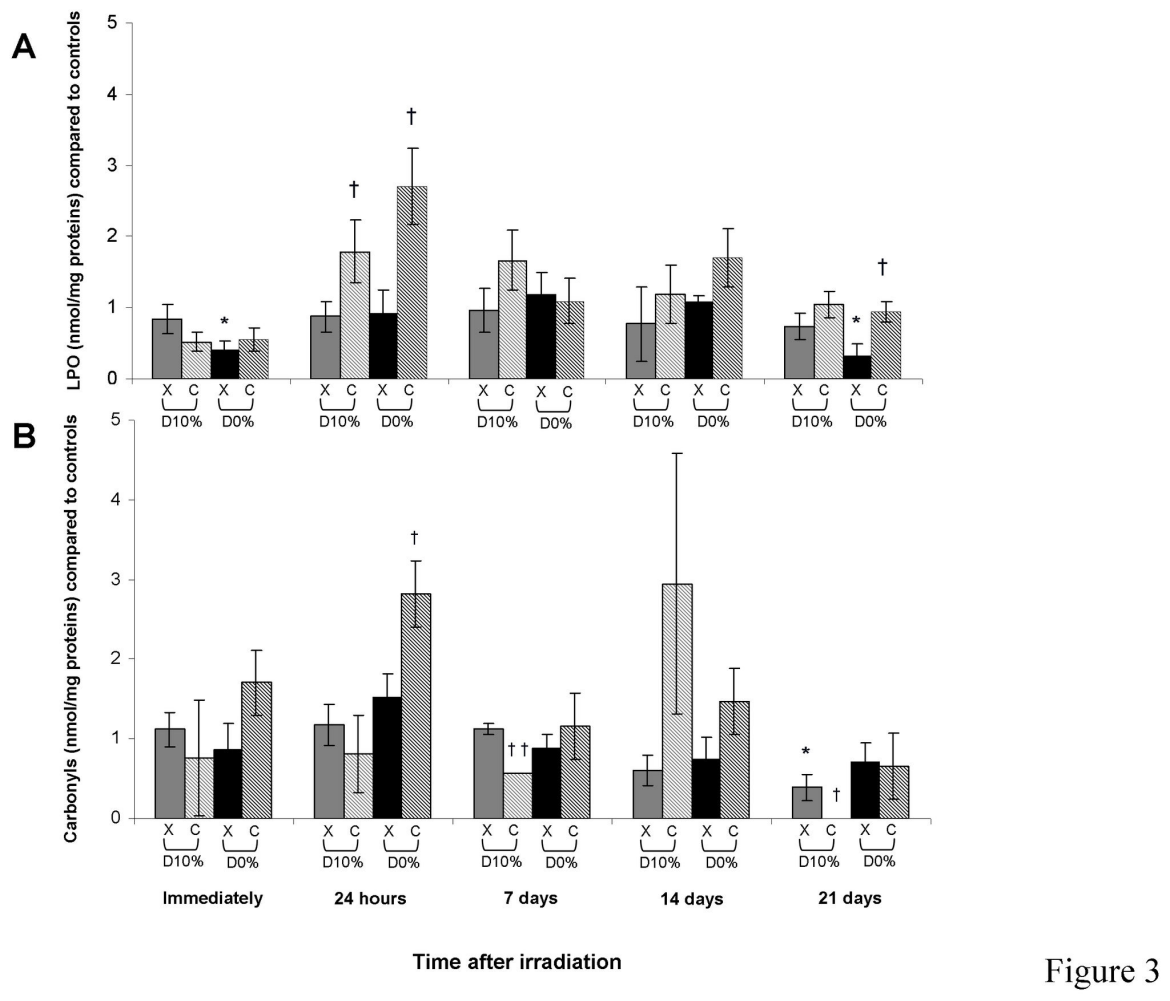
MDA and HAE (A) and protein carbonyl (B) concentrations in fibroblasts irradiated by X-rays or C-ions. Mean value of unirradiated cells was 0.54 ± 0.06 and 4.13 ± 1.03 nmol/mg protein (mean protein concentration in unirradiated cells = 3.35 ± 0.09 mg/mL), respectively.

ROS lead to polyunsaturated fatty acid peroxidation. Lipid hydroperoxides are degraded mainly into MDA and HAEs, which react in a covalent manner with proteins and inactivate them. MDA and HAE lipid peroxidation products were unchanged after X-ray irradiation except for D_0%_ immediately and at day 21 (Figure 3A). After C-ion irradiation, an increase was mainly observed at day 1 with an RBE_10%_ of 2.05. Carbonyl groups resulting from protein oxidation were quantified (Figure 3B). Their quantity was unchanged by X-rays except for a decrease for D_10%_ at day 21. After C-ion exposure, a 2.8-fold increase was observed at day 1 for D_0%_. For D_10%_, a 1.8-fold decrease was observed at day 7 and the concentration of carbonyl groups finally reached ~0 at day 21. A non-significant increase for D_10%_ and D_0%_ occurred 14 days after C-ion irradiation with an RBE_10%_ of 4.83.

### Antioxidant enzyme activities

**Figure 4:**
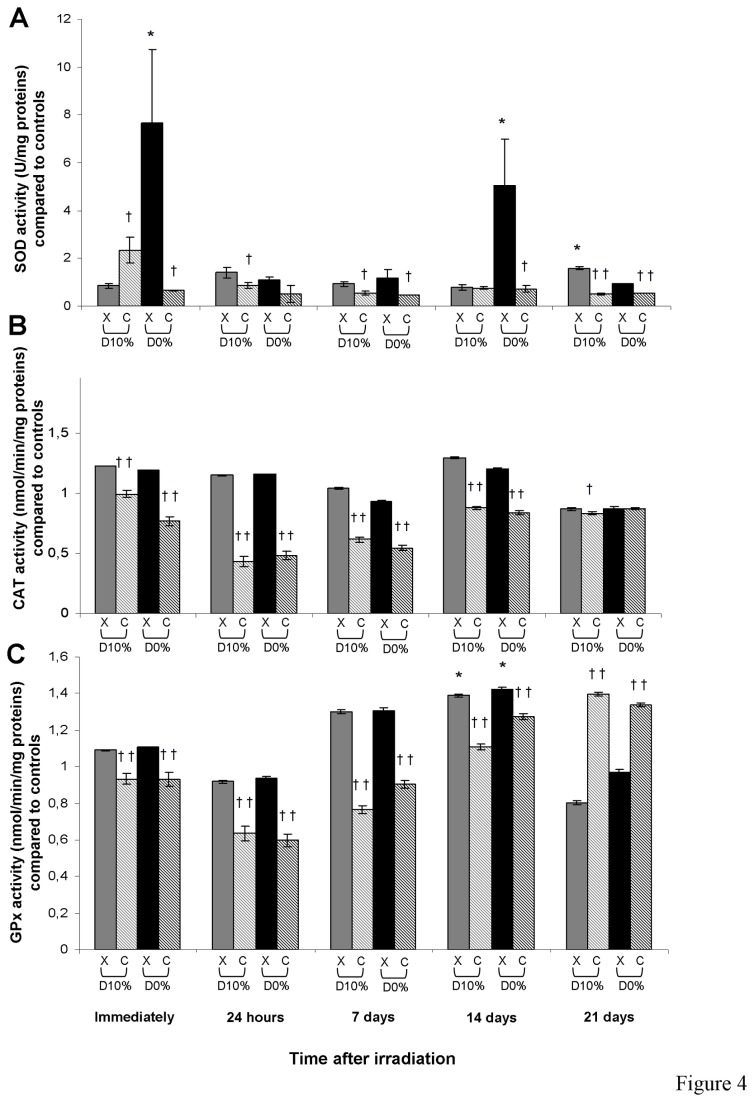
SOD (A), catalase (B) and GPx (C) activities in fibroblasts irradiated by X-rays or C-ions. Mean value of unirradiated cells was 1.60 ± 0.32 U/mg protein, 20.18 ± 0.94 and 35.73 ± 3.08 nmol/min/mg protein (mean protein concentration in unirradiated cells = 3.35 ± 0.09 mg/mL), respectively.

The main three antioxidant enzyme activities were quantified. After X-rays, total SOD activity was increased immediately and at day 14 at D_0%_ (Figure 4A). After C-ion irradiation, SOD activity was decreased compared with X-rays (except for D_10%_ immediately after irradiation), with an RBE_10%_ of 0.58 at day 7. Catalase activity was unchanged after X-rays, but was decreased after C-ions compared with X-rays (except for D_0%_ at day 21), with an RBE_10%_ of 0.59 at day 7 (Figure 4B). Like catalase activity, GPx activity was unchanged after X-rays except for a slight significant increase at day 14, but was decreased after C-ions compared with X-rays (except at day 21 where an increase was observed), with an RBE_10%_ of 0.59 at day 7 (Figure 4C).

### Reduced and oxidized glutathione ratio

**Figure 5:**
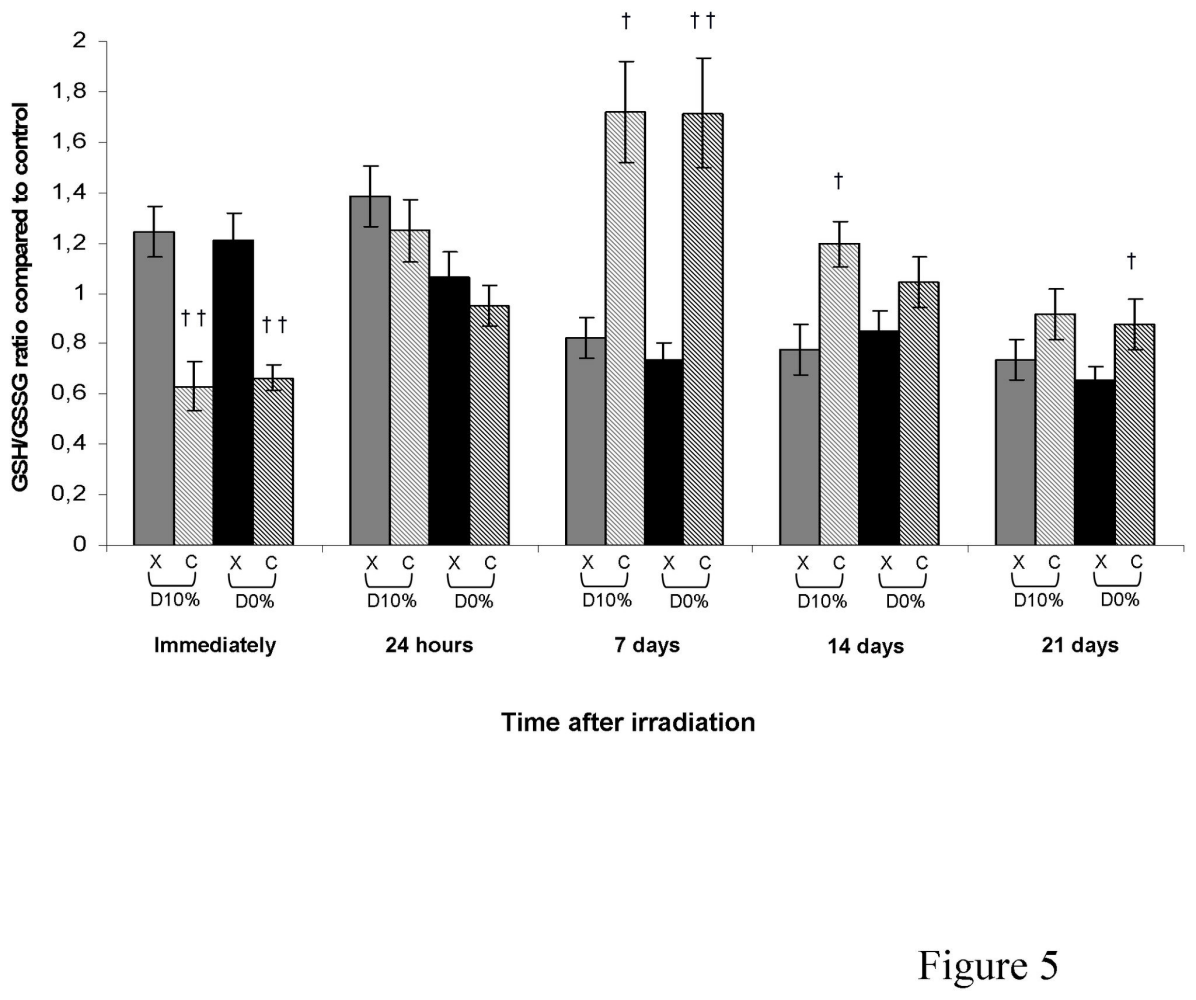
Reduced and oxidized glutathione ratio in fibroblasts irradiated by X-rays or C-ions. Mean value of unirradiated cells was 33.76 ± 5.80 with total GSH mean of 11.00 ± 1.48 µM/mg protein and GSSG mean of 0.33 ± 0.02 µM/mg protein (mean protein concentration in unirradiated cells = 3.35 ± 0.09 mg/mL).

Glutathione, which represents a major antioxidant defence system, is a tripeptide that can give an electron or hydrogen atom to a peroxide in an oxidation reaction catabolized by GPx. Glutathione is then in its reduced form (GSH) or its oxidized form (GSSG). GSSG must be reduced again to GSH by the action of glutathione reductase. The ratio GSH/GSSSG is usually used as a cellular indicator of redox potential as glutathione is responsible for the accumulation of hydrogen peroxide and the generation of severe OS. In X-ray irradiated fibroblasts, GSH/GSSG ratio was not significantly changed, whereas a decrease in comparison with X-rays was observed immediately after C-ion irradiation before an increase from day 7, with an RBE_10%_ of 2.09 at day 7 (Figure 5).

### TNF-α, IL-1β and IL-6 secretion

**Figure 6:**
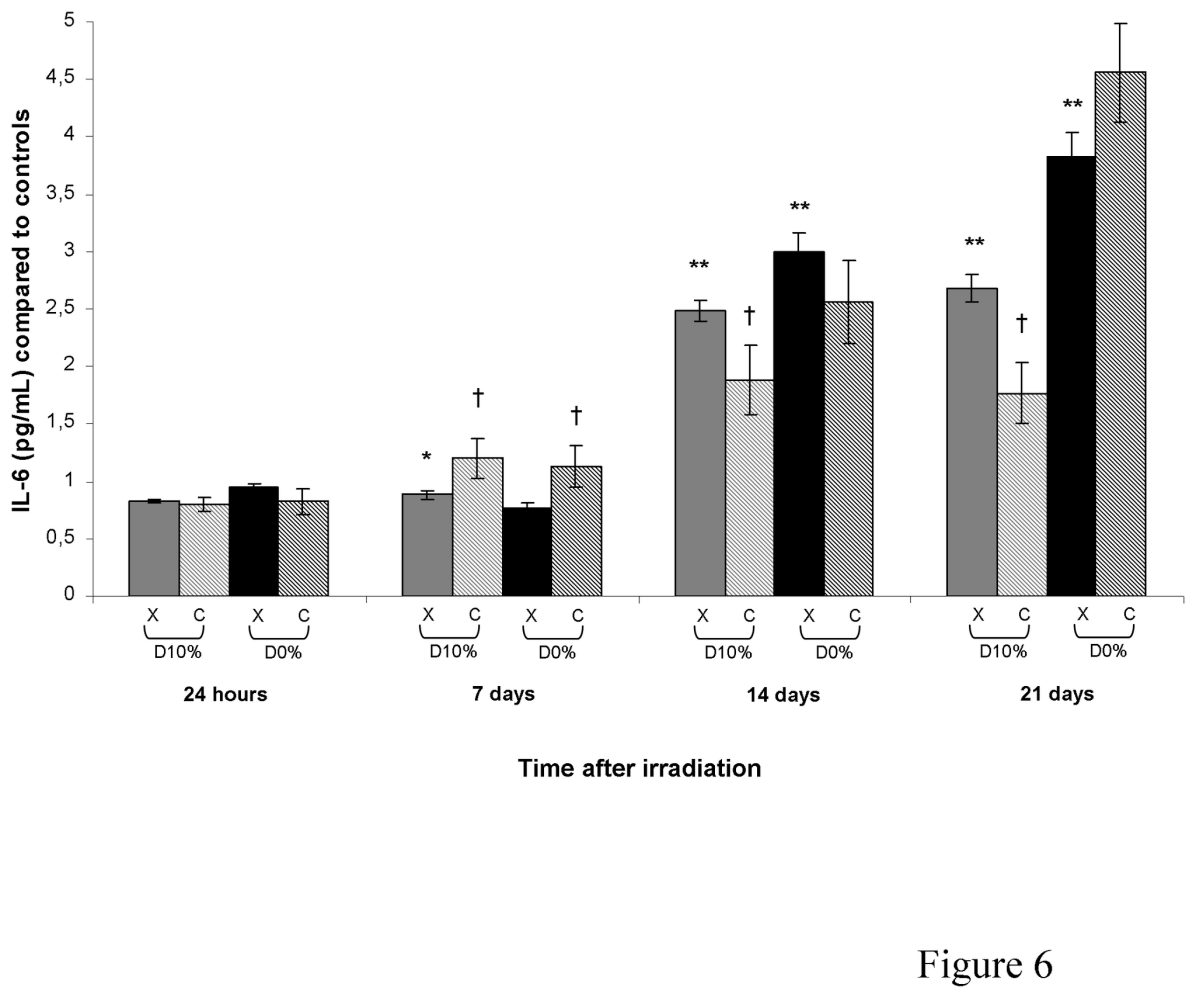
IL-6 concentration in supernatants of fibroblast cultures irradiated by X-rays or C-ions. Mean IL-6 concentration in unirradiated culture supernatants was 515.61 ± 21.67 pg/mL.

TNF-α, IL-1β and IL-6 are mediators of the inflammatory response and can be secreted by activated macrophages, T cells, smooth muscle cells and fibroblasts. IL-1β in culture supernatants could not be detected and TNF-α was unchanged after X-ray or C-ion irradiation (data not shown). An increase in IL-6 concentration occurred from day 14 after X-rays reaching a maximum at day 21 with a 2.7-fold increase for D_10%_ and a 3.8-fold increase for D_0%_ (Figure 6). After C-ions and in comparison with X-rays, an increase occurred at day 7 for D_10%_ and D_0%_ with an RBE_10%_ of 1.35 before a decrease on days 14 and 21 only for D_10%_, with an RBE_10%_ of 0.66.

## Discussion

In the present work, normal human skin fibroblasts were exposed to 33.6 keV/µm C-ion beams [16]. This LET corresponding to plateau-phase before SOBP in C-ion RT was adapted to our experiments on dermal fibroblasts as skin is the first organ exposed at the entrance of the beam. Skin complications were reported for the first time by Yanagi et al. [3] concerning patients treated with C-ions. Thirty-five patients treated for unresectable bone and soft tissue sarcoma by a dose escalation protocol were studied: 35 presented acute skin reactions and 27 developed late skin reactions. 

Survival curves and resulting radiobiological parameters showed a greater harmful effect of C-ions than of X-rays, with an RBE_37%_ of 4.77. Moreover, taking into account uncertainties concerning GANIL C-ion dosimetry as reported by Pautard et al. [18], the discrepancy between C-ions and X-rays may be greater than thought. Confluent fibroblasts were not as radiosensitive to X-rays when α and β values were low. In contrast, the fibroblast survival curve after C-ion irradiation almost followed a linear model with a high α value and a β value close to 0, suggesting that primary DNA damage was repaired with difficulty. Irradiation doses chosen for subsequent experiments corresponded to around D_10%_ and D_0%_. Chosen C-ion irradiation doses were in the range of 3.3 to 4.6 GyE/fraction used at NIRS and responsible for skin complications [3]. To understand the origins of this deleterious effect of C-ions compared with X-rays, we were interested in the OS pathway, which may explain the appearance of late cutaneous damage. 

In the first hour after irradiation, DNA damage was increased less after C-ions than after X-rays. C-ion damage was produced in clusters leading to smaller DNA fragments compared with X-rays. The comet assay takes into account the quantity of DNA in the comet tail so X-rays should produce larger fragments, thus increasing the measured OTMs. In this way, the alkaline comet assay may underestimate DNA damage produced by C-ions. Interestingly, an increase in DNA damage was observed 3 hours after C-ion irradiation. This increase could be induced by (i) new ROS production, (ii) secondary strand-breaks produced during the DNA repair process, as intermediate breaks before ligation, or (iii) DNA misrepair producing damage. As it is well known that DNA damage clusters may be wrongly repaired, the third hypothesis should be preferred. Moreover, the almost linear shape of the fibroblast survival curve after C-ion irradiation suggested DNA damage difficult to repair, which is in agreement with DNA damage clusters. 

In addition to DNA damage, ROS induced by X-rays and C-ions cause primary lesions that may affect lipids and proteins. Carbonyl groups result from protein oxidation. ROS can also induce polyunsaturated fatty acid peroxidation. Lipid hydroperoxides are degraded mainly into MDA and HAEs, which react covalently with and may inactivate proteins. Lipid peroxidation and protein carbonylation measurements were not greatly changed. The main endpoint occurred at day 1 with an increase in MDA and HAEs and in carbonyl values in fibroblasts irradiated by C-ions compared with X-rays. This early wave of lipid and protein degradation products could be reduced at later times due to lipid and protein repair.

Surprisingly, the three main antioxidant enzyme activities were decreased after C-ion irradiation. This decreased detoxifying capacity after C-ion irradiation suggests that antioxidant enzymes were not active after irradiation, due either to a transcription decrease or to an inhibition, except for GPx activity on day 21 suggesting a late increase in transcription perhaps due to the prolonged exposure to oxidative stress. GSH/GSSG ratio was increased from day 7 after C-ion irradiation compared with X-rays. This increase was not due to an increase in GSH synthesis as its amount was not much changed after irradiation except at day 21 where a strong decrease was observed after X-rays (data not shown). This GSH/GSSG increase could be linked to the decrease in the activity of GPx which uses GSH to detoxify peroxide. It seems worthwhile evaluating glutathione reductase activity as this enzyme recycles GSSG into GSH. Overall, our results suggest that C-ion irradiation induced an imbalance between ROS production and ROS detoxification processes leading to persistent oxidative lesions in normal skin fibroblasts.

Finally, we investigated expression of inflammatory mediators, which is generally linked to ROS production. Surprisingly, no significant changes in TNF-α expression were observed after C-ion *vs.* X-ray irradiation. Moreover, IL1-β levels were undetectable. IL-6 level increases after both types of irradiation according to the literature [19]. Interestingly, IL-6 concentration was increased more on day 7 after C-ions than after X-rays. However, IL-6 level after D_10%_ C-ion irradiation was lower on days 14 and 21 compared with X-rays. In this way, inflammatory pathways did not seem to be strongly involved in the deleterious effects observed after C-ion irradiation of skin fibroblasts, except for IL-6 on day 7. 

Our experiments suggest that macromolecular damage was greatly increased and that antioxidant defences were much decreased during the first three weeks after C-ion compared with X-ray irradiation. Taken together, these data could explain, at least in part, the late cutaneous and sub-cutaneous complications reported by Yanagi et al. [3]. Further work is needed to understand the reasons for this increase in OS in normal human skin fibroblasts exposed to C-ions. Studies on gene regulation of oxidative metabolism, DNA damage and repair, and senescence pathways are already in progress. Studies on other cell types (haematopoietic stem/progenitor cells, oral squamous cell carcinoma) have been reported [14,20]. But C-ion irradiation was not compared with X-rays and values of interest were not related to protein concentration.
